# Si-RNA mediated knockdown of CELF1 gene suppressed the proliferation of human lung cancer cells

**DOI:** 10.1186/1475-2867-13-115

**Published:** 2013-11-15

**Authors:** Li-Na Wu, Yi-Jun Xue, Li-Jian Zhang, Xue-Mei Ma, Jin-Feng Chen

**Affiliations:** 1 Key laboratory of Carcinogenesis and Translational Research (Ministry of Education), Central Laboratory, Peking University Cancer Hospital & Institute, Beijing 100142, China; 2 The College of Life Science and Bio-engineering, Beijing University of Technology, Beijing 100022, People’s Republic of China; 3 Key laboratory of Carcinogenesis and Translational Research (Ministry of Education), Department of Thoracic Surgery II, Peking University Cancer Hospital & Institute, Beijing 100142, China

**Keywords:** Lung cancer, CELF1 gene, Proliferation

## Abstract

**Background:**

Lung cancer is the leading cause of cancer-related death in the world, with metastasis as the main reason for the mortality. CELF1 is an RNA-binding protein controlling the post-transcriptional regulation of genes related to cell survival. As yet, there is little knowledge of CELF1 expression and biological function in lung cancer. This study investigated the expression levels of CELF1 in lung cancer tissues and the biological function of CELF1 in lung cancer cells.

**Methods:**

CELF1 mRNA expression was determined in lung cancer and normal tissues, and the relationship between the expression level of CELF1 and clinicopathological parameters was evaluated. The biological function of CELF1 in A549 and H1299 lung cancer cell lines growth was examined.

**Results:**

The expression of CELF1 was higher in human lung cancer tissues compared with the normal lung tissue. Lentiviral-mediated transfection of CELF1 siRNA effectively silenced the expression of CELF1 in both A549 and H1299 cells. Moreover, CELF1 knockdown markedly reduced the survival rate of lung cancer cells. Colony formation assays revealed a reduction in the number and size of lung cancer cell colonies from CELF1 knockdown.

**Conclusion:**

These results indicated that CELF1 may have significant roles in the progression of lung cancer, and suggested that siRNA mediated silencing of CELF1 could be an effective tool in lung cancer treatment.

## Introduction

Lung cancer is one of the leading causes of cancer-related deaths worldwide [1]. Studies have shown that the genes and target proteins involved in lung cancer function in cell proliferation [2], apoptosis [3], and angiogenesis [4] pathways. Identifying a mechanism that inhibits the growth of lung cancer metastasis would be useful in developing potential treatments for lung cancer. Veale D et al. first reported the epidermal growth factor receptor (EGFR) was associated with spread of human non-small cell lung cancer and might be a potential therapeutic target in many carcinomas [5]. Now, the EGFR superfamily is well known to promote cancer cell growth, and has become a therapeutic target for lung cancer and changed the lung cancer treatment model. By exploring new cancer-related genes and clearly identifying the roles of these genes in tumor development and progression, not only can we obtain a deeper understanding of the nature of tumors, but we can also discover new tumor therapeutic targets.

The CELF (CUGBP and Etr-like factors) family proteins are major sequence-specific RNA binding proteins that control alternative splicing and mRNA translation and stability [6,7]. Some reports have demonstrated that CELF1 protein regulates pre-mRNA alternative splicing and is involved in mRNA editing and translation [8-10]. Whether the expression of the CELF1 gene is related to the proliferation of human lung cancer has not been investigated.

Here we investigated the relationship between CELF1 expression and lung cancer clinicopathological factors at the RNA level and clarified the physiological impact of CELF1 on lung cancer cell growth at the cellular level.

## Results

### Expression of CELF1 in lung cancer tissues

**Figure 1:**
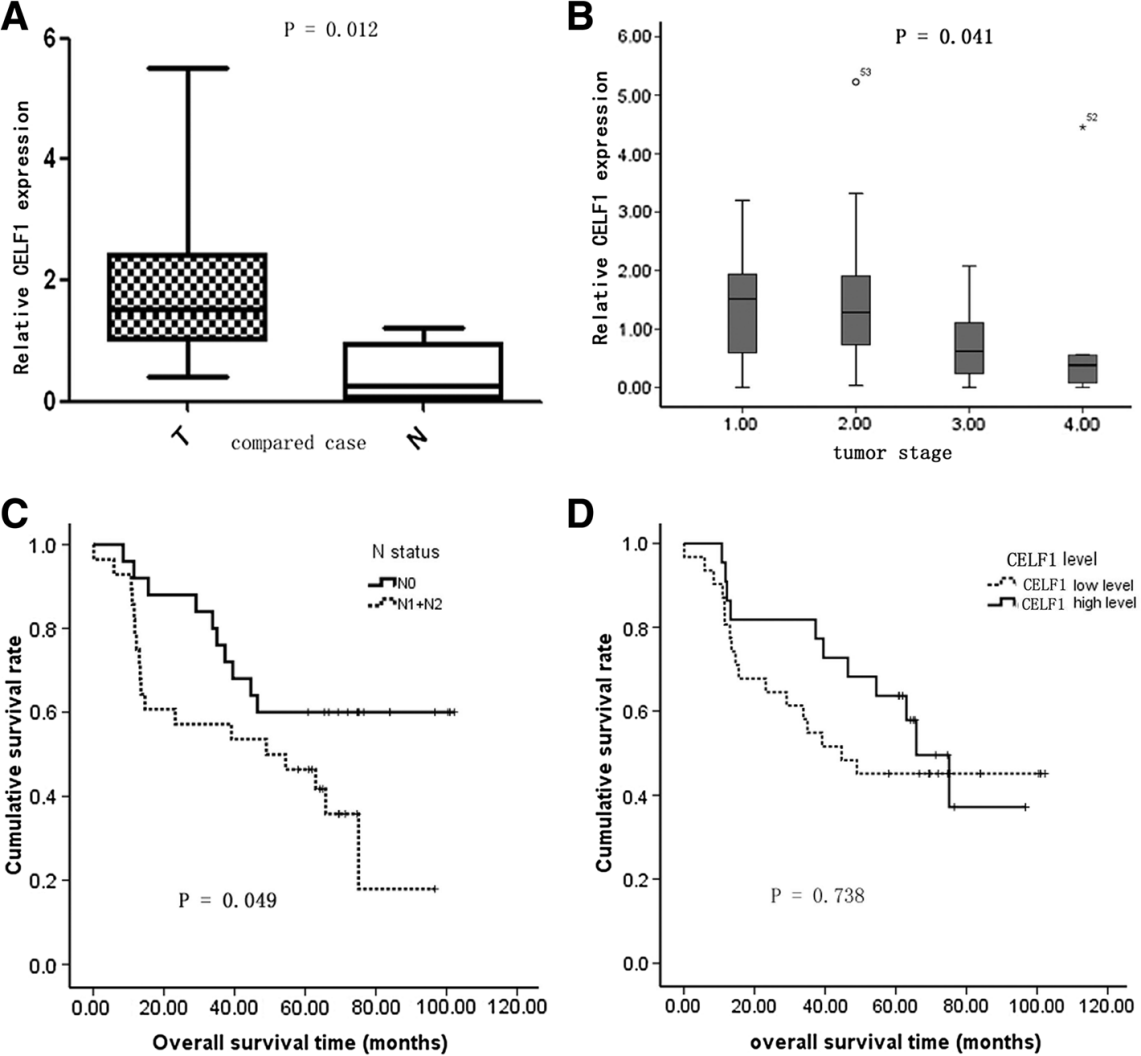
**Expression levels of CELF1 in lung cancer tissues. (A)** Expression levels of CELF1 in 10 lung cancer tissues compared with normal lung tissues analyzed by real time PCR. **(B)** Expression of CELF1 in lung cancers at different stages analyzed by real time PCR. **(C)** Comparison of the survival rate of patients with N1 and N2 lymph node metastasis and without lymph node metastasis. **(D)** Comparison of the survival rate with levels of CELF1 expression. The values represents the mean from three independent experiments; bars represent SD. *P < 0.05, **P < 0.01 compared with controls.

To evaluate the levels of CELF1 expression in lung cancer tissues and normal tissues, real time PCR was performed in 10 lung cancer tissues and 10 normal tissues. Results showed that the relative expression levels of CELF1 were higher in lung cancer tissues compared with the normal tissues (Figure 1A and Table 1). Moreover, the expression levels of CELF1 in lung cancer tumors varied depending on tumor grade. CELF1 expression levels were higher in low-grade cancers compared with high-grade cancers (Figure 1B). A higher expression level of CELF1 was observed in patients with the increase of T stage, indicating that when tumors begin to grow larger, gradually some other mechanism(s) may play a more important role to promote cancer cell growth, so the CELF1 expression level had some decrease. Furthermore, comparison of the survival rate of patients with N1 and N2 lymph node metastasis and without lymph node metastasis showed that the survival rates were significantly higher in the absence of lymph node metastasis (Figure 1C). In comparing the survival rate with CELF1 expression levels, no significant difference in survival rate was observed between patients with higher CELF1 expression and lower expression (Figure 1D). Together these data indicate that CELF1 expression is not related to postoperative survival in lung cancer patients.

**Table 1:** Expression level of CELF1 in lung carcinoma and relationship to clinicopathological parameters

**Variable**	**Case no.**	**CELF1 expression (RQ: 2**^**-ΔΔCT**^**)**		**P value**
		**Median**	**Mean ± SEM**	
Gender				
Male	41	1.1090	1.2458 ± 0.9115	0.183
Female	12	0.7252	0.8938 ± 103.53	
Age				
≤ 60.5	32	0.9000	1.0103 ± 0.8746	0.118
> 60.5	21	0.1164	1.4035 ± 1.4948	
Venous invasion				
Negative	40	1.0274	1.2302 ± 1.2217	0.251
Positive	13	0.5492	0.9687 ± 0.9781	
T stage				
T1	7	1.5105	1.396 ± 1.1033	**0.041**
T2	21	1.2776	1.5178 ± 1.2898	
T3	17	0.6242	0.8040 ± 0.7079	
T4	8	0.3816	0.8109 ± 1.4910	
N stage				
N0	25	0.6242	0.9983 ± 1.1184	0.251
N1	11	1.3165	1.4731 ± 1.4334	
N2	17	1.1164	1.2142 ± 1.0611	
Lymph node status				
N0	25	0.6242	0.9983 ± 1.1184	0.164
N1 + N2	28	1.2321	1.3159 ± 1.2020	
TNM stage				
Ia	4	1.2109	1.4055 ± 1.3481	0.089
Ib	6	1.2500	1.4397 ± 0.9834	
IIa	5	1.3322	2.1861 ± 1.7131	
IIb	13	0.6242	0.7331 ± 0.5595	
IIIa	23	0.6624	1.1535 ± 1.2526	
IIIb	2	0.2747	0.2747 ± 0.3882	
Differentiation				
Well	6	1.0545	0.8122 ± 0.5568	0.256
Moderate	28	0.9000	1.1866 ± 1.1315	
Poor	19	1.0548	1.2476 ± 1.3613	
Compared case				
Tumor tissue	10	1.5263	1.9005 ± 1.4382	**0.012**
Normal tissue	10	0.2509	0.4579 ± 0.4761	

### Effect of CELF1 siRNA on the expression levels of CELF1 in lung cancer cells

**Figure 2:**
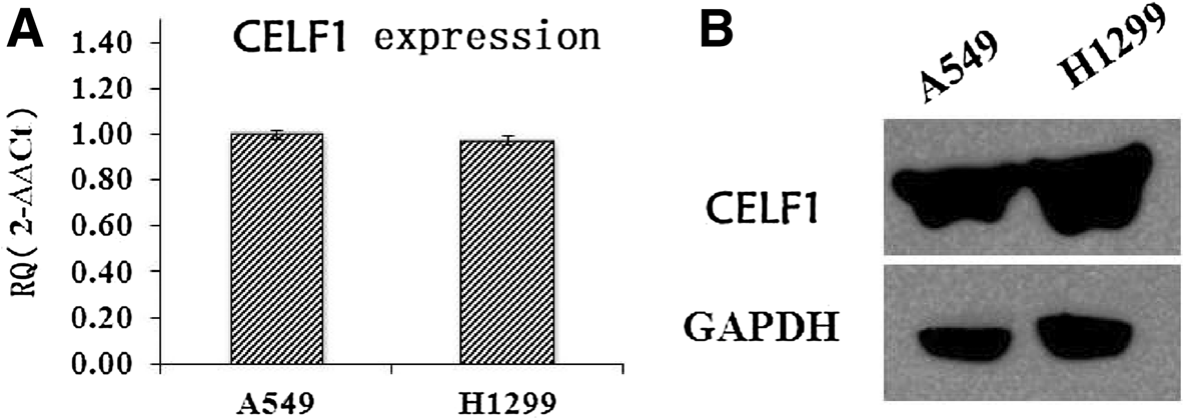
**Expression levels of CELF1 in lung cancer cells. (A)**. Expression levels of CELF1 in A549 and H1299 cells analyzed by real time PCR. **(B)**. Protein expression levels of CELF1 in A549 and H1299 cells analyzed by western blotting. The values represents the mean from three independent experiments; bars represent SD. *P < 0.05, **P < 0.01 compared with controls.

**Figure 3:**
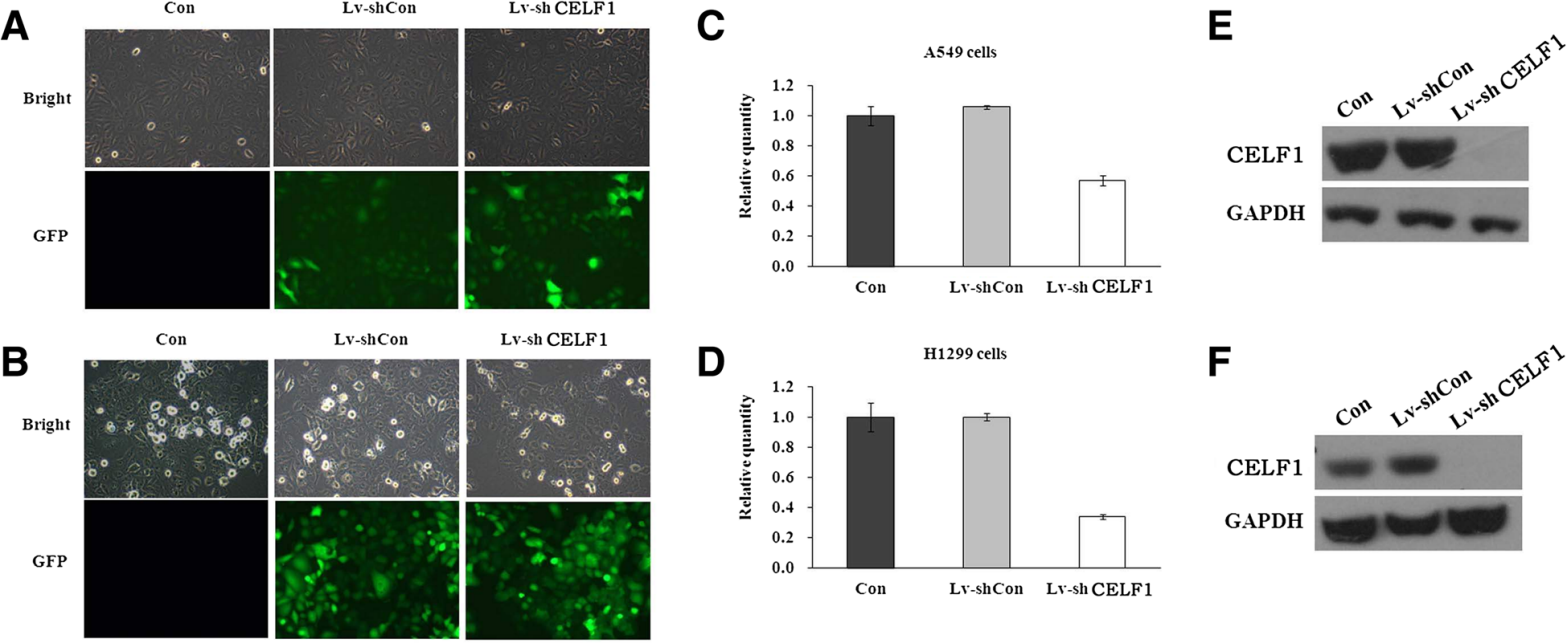
**Knockdown of CELF1 gene in lung cancer cells. (A, B)** Infection efficiency of lentivirus containing CELF1 siRNA in A549 **(A)** and H1299 **(B)** cells. The efficiency was analyzed by fluorescence microscopic analysis of infected cells. **(C, D)** The comparative gene expression levels of CELF1 in normal cells, CELF1-silenced cells and non-silencing lentivirus infected cells analyzed by real time PCR. **(E, F)**. The comparative expression levels of CELF1 protein in normal cells, CELF1-silenced cells and non-silencing lentivirus infected cells analyzed by western blotting. The values represent the mean from three independent experiments; bars represent SD.

Next, CELF1 mRNA and protein levels in A549 and H1299 lung cancer cells were evaluated by real time PCR and western blot, respectively. As shown in Figure 2, both gene and protein expression of CELF1 were detected in A549 and H1299 cells. The CELF1expressing lung cancer cells were then infected with lentivirus containing CELF1 shRNA or non-silencing control shRNA, and successful infection was confirmed by green fluorescence of infected cells (Figure 3A and B). Fluorescence analysis showed that the lentiviral infection rate was higher in H1299 cells than in A549 cells. Infection of cells with lentivirus containing CELF1 shRNA significantly reduced CELF1 gene and protein expression levels in both A549 and H1299 cells (Figure 3C-F). In contrast, the non-silencing siRNA infection had no effect on CELF1 levels, confirming that CELF1 expression levels were reduced specifically from CELF1 siRNA.

### Effect of CELF1 knockdown on the survival of lung cancer cells

**Figure 4:**
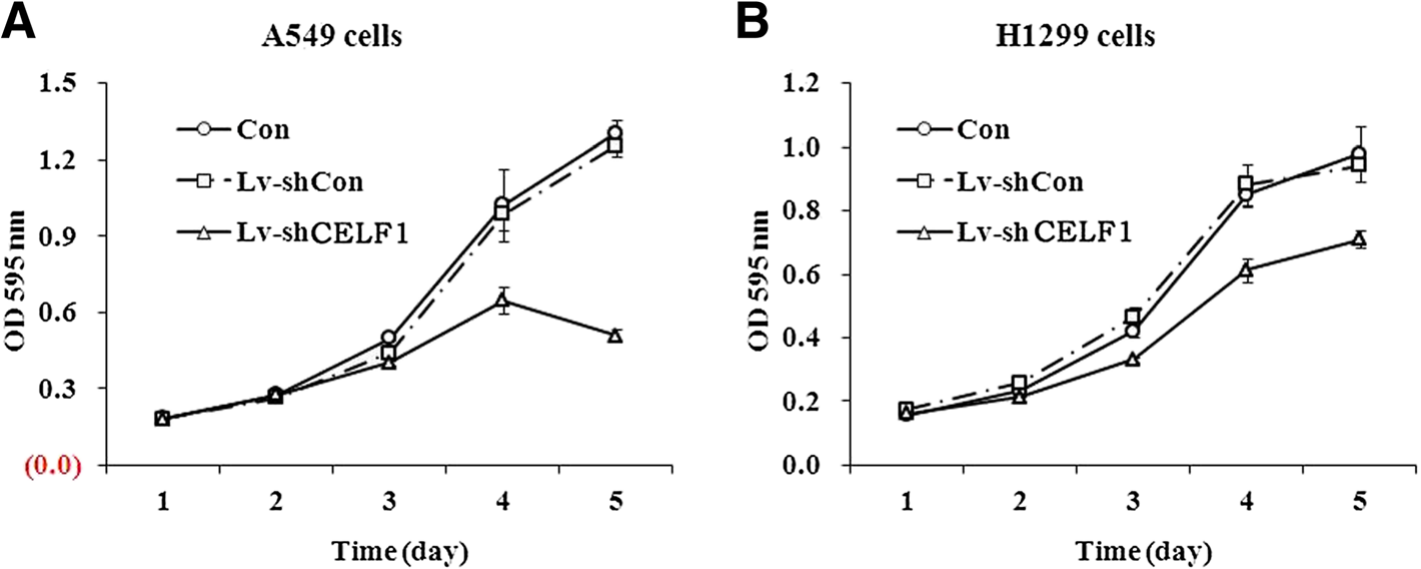
**Effect of CELF1 gene knockdown on the proliferation of lung cancer cells.** Proliferation of A549 **(A)** and A1299 **(B)** cells were treated as indicated and measured by MTT assay. The values represents the mean from three independent experiments; bars represent SD. ***P < 0.001 compared with controls.

The effect of CELF1 knockdown on lung cancer cell survival was analyzed using a MTT assay performed over a five-day time course. In both cell lines, significant differences in cell survival were not observed until three days after infection. From the 4^th^ day of infection onwards, significant differences in cell survival were observed in both cell lines. The survival rate of CELF1 knockdown cells was markedly (*P* < 0.0001) reduced at the 5^th^ day of infection compared with the non-infected and control siRNA infected A549 and H1299 cells (Figure 4). The reduction in cell survival due to CELF1 knockdown was higher in A549 cells than in H1299 cells.

### Effect of CELF1 knockdown on the colony forming ability of lung cancer cells

**Figure 5:**
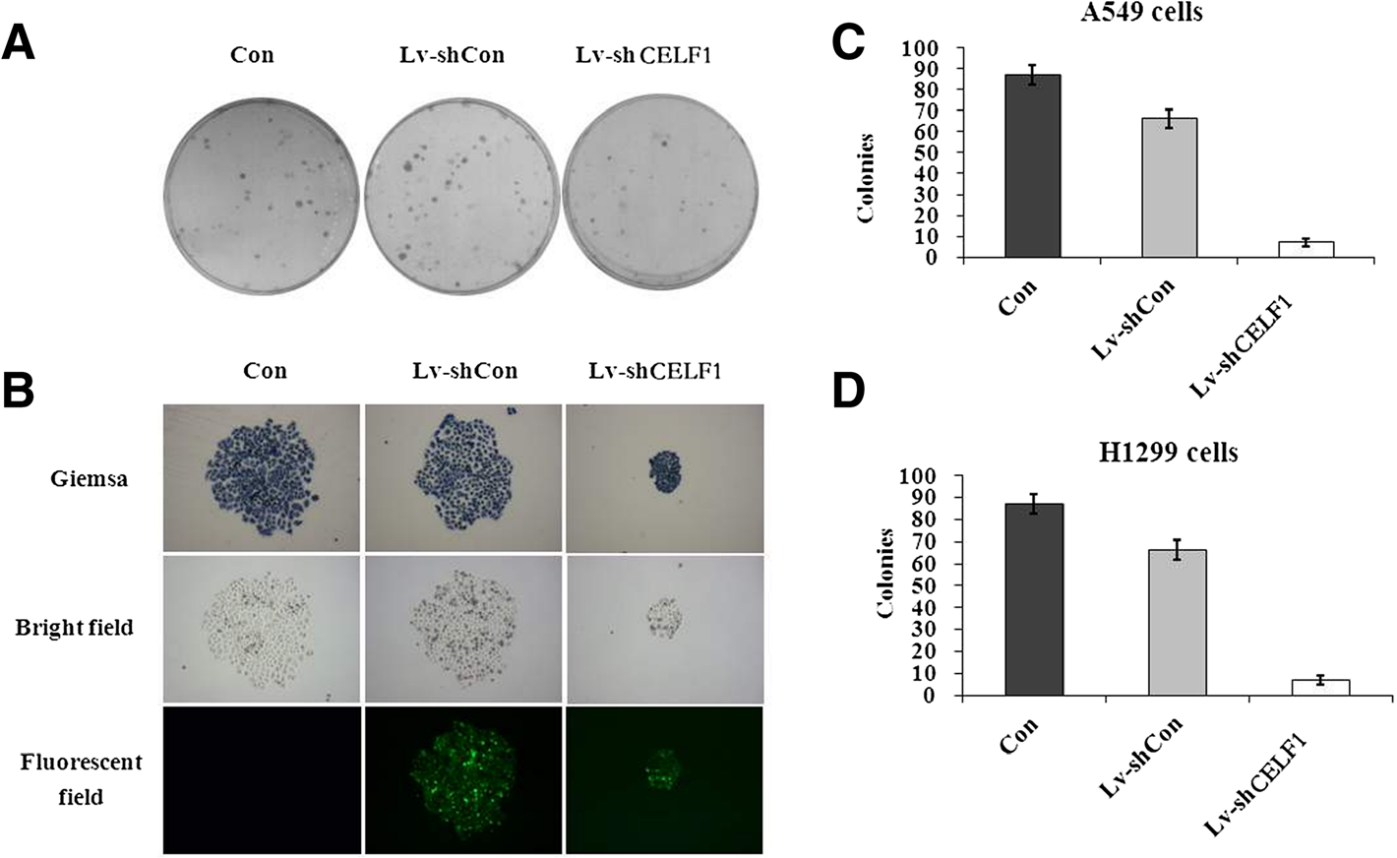
**Effect of CELF1 gene knockdown on the colony forming ability of lung cancer cells. (A, B)** The cancer cell colonies under light, bright field and fluorescence microscopy. The colonies were stained with Giemsa staining. The images show comparison of the size of colonies in normal, CELF1-silenced and non-silenced lung cancer cells. **(C, D)** The number of colonies in normal, CELF1-silenced and non-silenced lung cancer cells. The values represents the mean from three independent experiments; bars represent SD. **P < 0.01, ***P < 0.001 compared with controls.

Lung cancer cells tend to form large cell colonies while in culture; therefore, we next evaluated the effect of CELF1 silencing on the colony forming ability of lung cancer cells. As shown in Figure 5A and B, the size of colonies in CELF1-silenced A549 and H1299 cells was markedly reduced compared with the control groups. Similarly, the number of colonies was also significantly (*P* < 0.001) reduced with the CELF1 gene knockdown. Together these results indicate that CELF1 plays an important role in the growth progression of lung cancer cells.

## Discussion

With increasing mortality rates, lung carcinoma has already become the leading cause of cancer mortality in the world [11]. Many genes are subjected to post-transcriptional regulation via control of the rate of mRNA turnover for transcripts bearing destabilizing *cis*-elements [12]. Among the very few regulatory factors identified thus far, CELF1 regulates post-transcriptional gene expression by facilitating alternative splicing, translation [13], and mRNA degradation, and it functions by binding directly to RNA [14]. Rattenbacher et al. identified the CELF1 gene and its target proteins as a critical posttranscriptional regulatory network that may play a role in the development of cancer [8]. In addition to reports of involvement in breast cancer and leukemia development, the CELF1 gene may also play a significant role in tumorigenesis and the deterioration of certain tumors [15], which is also confirmed by the results in our present study.

Timchenko et al. first identified CELF1 function in the regulation of translation of C/EBP beta isoforms [6]. Subsequent research demonstrated that members of this protein family regulate pre-mRNA alternative splicing and may also be involved in mRNA editing and translation [16]. The CELF1 gene may play a role in myotonic dystrophy type 1 (DM1) via interactions with the dystrophia myotonica-protein kinase (DMPK) gene [10]. A previous report identified a correlation between the expression of CELF1 and human lung cancer [17]. However, the cellular mechanism underlying how the CELF1 gene causes this phenomenon has not been clarified. Together these findings implicate possible involvement of the CELF1 gene in cell growth. So far there is no literature reporting the biological function of CELF1 gene in lung cancer cell. We speculate that CELF1 may also play an important role in lung cancer proliferation.

Our research primarily focused on the effect of CELF1 knockdown on the viability of lung cancer cells. As shown in the results, the expression of CELF1 was higher in human lung cancer tissues compared with normal tissues. Moreover, A549 and H1299 lung cancer cells also exhibited CELF1 expression in mRNA and protein level. Lentiviral-mediated delivery of CELF1 silencing siRNA significantly inhibited these upregulated levels of CELF1 expression, demonstrating that the CELF1 knockdown method was successful. Further *in vivo* studies should be performed to confirm the use of this siRNA method as a potential therapeutic tool.

Interestingly, upon knockdown of CELF1, the survival rates and colony forming ability of lung cancer cells were markedly reduced, indicating pivotal roles of CELF1 in the survival of lung cancer cells. Reports in the literature have suggested that upregulation of CELF1 increased the turnover of oncogenes related to the proliferation of lung cancer cells [7,9,18]. Hence, in the absence of CELF1, the turnover of possible oncogenes could presumably decrease, consistent with the cancer cells showing decreased capacity of proliferation and colony formation. Our study showed that CELF1 is overexpressed in lung cancer tissue on RNA level compared with the normal lung tissue and tumor grades had relationship with CELF1 expression level, which is line with the hypothesis mentioned above.

From these results, we can conclude that CELF1 can affect the growth of lung cancer cells and plays an important role in the tumor development process. Further research on the molecular mechanisms of the CELF1 gene is required, particularly in identifying CELF1-interacting proteins, elucidating the molecular mechanisms underlying its biological effects, and determining whether it plays a guiding role in the treatment of lung cancer.

## Conclusion

In summary, CELF1 may have significant roles in the progression of lung carcinoma. The CELF1 siRNA method has emerged as a potentially powerful tool for cancer therapeutics in silencing genes responsible for cancer progression and tumorigenesis.

## Materials and methods

### Human specimens and reagents

Fifty-three pulmonary cancer samples of fresh frozen tissue were acquired from the Department of Thoracic Surgery, Beijing cancer hospital, under approval from the Ethical Committee. Written consent statements were obtained from all patients before operation. None of the patients received any neoadjuvant therapy prior to surgery. The tissues were collected immediately after surgical resection at the Beijing Cancer Hospital and stored at the Tissue Bank of Peking University Oncology School. Clinicopathological characteristics of the tumors were defined according to the TNM staging system criteria of UICC. Clinicopathological factors are shown in Table 1.

AgeI, EcoRI, and SYBRGreen Master Mix Kits were purchased from TaKaRa (Dalian, China). pHelper1.0, pHelper 2.0, and pGCSIL-GFP plasmids were purchased from Genechem Co. Ltd (Shanghai, China). The RNeasy Midi Kit was from Qiagen (Valencia, CA, USA). Dulbecco’s modified Eagle’s medium (DMEM), Roswell Park Memorial Institute 1640 (RPMI 1640) and fetal bovine serum (FBS) were obtained from Hyclone (Logan, UT, USA). Lipofectamine2000, TRIzol and SuperScriptII reverse transcriptase were purchased from Invitrogen (Carlsbad, CA, USA). All other chemicals were obtained from Sigma (St. Louis, MO, USA). The following antibodies were obtained from Santa Cruz: anti-CELF1 (1:1000 dilution), anti-GAPDH (glyceraldehyde-3-phosphate dehydrogenase, 1:3000 dilution) and anti-mouse HRP (1:5000 dilution).

### Cell culture

Human lung cancer cells (A549 and H1299) and human embryonic kidney (HEK) 293 T cell lines were obtained from the cell bank of Shanghai Institute of Cell Biology. A549 and 293 T cells were maintained in DMEM supplemented with 10% heat-inactivated FBS and penicillin/streptomycin at 37°C in a humidified atmosphere of 5% CO_2_. H1299 cells were maintained in RPMI 1640 medium supplemented with 10% heat-inactivated FBS and penicillin/streptomycin at 37°C in a humidified atmosphere of 5% CO_2_.

### Construction of CELF1 shRNA-containing lentivirus and infection

The sequences of CELF1 siRNA and non-silencing control siRNA were 5′-CTAGCCGGGATTGAAGAATGCCGGATATTCAAGAGATATCCGGCATTCTTCAATCTTTTTAAT-3′ and 5′-CTAGCCCGGTTCTCCGAACGTGTCACGTATCTCGAGATACGTGACACGTTCGGAGAATTTTTTTAAT-3′, respectively. The nucleotide sequences were inserted into the plasmid through the pFH-L vector (Shanghai Hollybio, China) and the generated lentiviral-based shRNA-expressing vectors were confirmed by DNA sequencing. Lentiviruses were generated by transfection of 293 T cells at 80% confluence with generated plasmids. The cells were starved for 2 h before transfection, and pFH-L-shCELF1 or -shCTRL and the packaging vector carriers pVSVG-I and PCMVΔR8.92 were transfected into cells using Lipofectamine 2000. The supernatant was collected 48 h after transfection and lentiviral particles were harvested by ultracentrifugation (4000 *g*) at 4°C for 10 min. The collected virus particles were filtered through a 45 μM filter and the filtrate was centrifuged (4000 *g* at 4°C) for 15 min to collect the viral concentrate.

A549 and H1299 cancer cells were then infected with CELF1 shRNA- or control shRNA-expressing lentiviruses at a MOI of 30 for A549 cells or MOI of 15 for H1299 cells. The cells were seeded (5 × 10^4^ cells per well) in six-well plates, and after 24 h of incubation, the culture medium was replaced with Opti-MEM medium containing the appropriate amount of the virus. The cells were then incubated with the virus for another 48 h. Successful transfection was confirmed by observation through a fluorescence microscope (Leica, Germany) for expression of green fluorescence protein.

### Real-time PCR analysis

RNA was obtained using the Total RNA Isolation Reagent (ABgene™) according to the manufacturer’s instructions (Abgene, Surrey, UK). Total RNA was converted to cDNA using DuraScript™, a commercial reverse transcription kit from Sigma Aldrich. Real-time quantitative PCR analysis for the RNA extracted from lung cancer tissues and cultured lung cancer cells was performed using the SYBR Green Master Mix Kit (Applied Biosystems, Foster City, CA). In brief, each PCR reaction mixture contained 10 μl of 2 × SYBR Green Master Mix, 0.4 μl of sense and antisense primers (2.5 μM) and 10 ng of cDNA. Reactions were run for 40 cycles, including denaturation at 95°C for 10 min and annealing at 60°C for 1 min in a total volume of 20 μl using an ABI 7500 Real Time PCR platform. The primer sequences for PCR amplification of the CELF1 gene were 5′-ACCTGTTCATCTACCACCTG-3′ and 5′-GGCTTGCTGTCATTCTTCG-3′. Primer sequences for the internal control GAPDH were 5′-GACCCCTTCATTGACCTCAAC-3′ and 5′-CTTCTCCATGGTGGTGAAGA-3′. Relative gene expression levels were calculated using 2-ΔΔCT analysis.

### Western blot analysis

A549 and H1299 cells were infected with the lentivirus containing CELF1 shRNA and control shRNA for five days. The cells were then washed with cold PBS and lysed with radio-immune precipitation assay (RIPA) buffer (50 mM Tris, pH 7.5, 150 mM NaCl, 1% NP-40, 0.5% sodium deoxycholate, 0.1% SDS) containing phenylmethylsulfonyl fluoride (PMSF) (1 mM) and protease inhibitors (2 μg/ml; Protease Inhibitor Cocktail Set III, Calbiochem) on ice for 30 min. The supernatant was collected after centrifuging the cell lysate (12,000 × *g* for 15 min) and the protein content was measured by the Lowry method. The protein concentration of each sample was adjusted to 2 μg/μl and a 20 μl volume was mixed with 2 × SDS sample buffer (100 mM Tris–HCl, pH 6.8, 10 mM EDTA, 4% SDS, 10% glycine) and separated by electrophoresis on a 10% SDS-PAGE gel at 50 V for 3 h. The gel was transferred to a PVDF membrane at 300 mA for 1.5 h, and proteins were detected after primary antibody treatment overnight at 4°C and secondary antibody treatment for 2 h at room temperature using an Amersham ECL kit (GE Healthcare, UK) and exposure to X-ray film. The bands obtained were quantified with an Image Quant densitometric scanner (Molecular Dynamics, Amersham Biosciences).

### MTT analysis

For the cell viability analysis, A549 and H1299 cells were first seeded (2 × 10^3^ cells/well) into a 96-well plate and infected with CELF1 silencing or non-silencing siRNA-containing lentivirus for 72 h. Following infection, 20 μl of MTT solution (5 mg/ml) was added to each well and cells were incubated at 37°C for 4 h. The medium and MTT from the wells was removed and 200 μl of DMSO was added to each well. The optical density was measured using a microplate reader at 490 nm. Experiments were performed in triplicate.

### Colony formation assay

Lung cancer cells seeded in six-well plates (2 × 10^2^ cells/well) were infected with CELF1 silencing and non-silencing siRNA-containing lentivirus for 72 h. The cells were continuously incubated, and medium was replaced with new medium every three days until 8 days of culture. The cells were then washed with PBS and fixed with 4% paraformaldehyde. The fixed cells were stained with freshly prepared diluted Giemsa stain for 20 min. The cells were washed with double distilled water and colonies were counted using a fluorescence microscope.

## Consent

Written informed consent was obtained from the patients before operation.

## Competing interests

The authors declare that they have no competing interests.

## Authors’ contributions

LNW and YJX contributed equally to the study design, experimental work, data analysis and preparation of the manuscript. JFC, LJZ and XMM participated in the design of the study and performed the statistical analysis. JFC conceived of the study, and participated in its design and coordination and helped to draft the manuscript. All authors read and approved the final manuscript.

## References

[B1] Parkin DM Pisani P Ferlay J Global cancer statistics CA: Cancer J Clin 1999 49 1 33 64 31 10.3322/canjclin.49.1.33 10200776

[B2] Caldon CE Lee CS Sutherland RL Musgrove EA Wilms’ tumor protein 1: an early target of progestin regulation in T-47D breast cancer cells that modulates proliferation and differentiation Oncogene 2008 27 1 126 138 10.1038/sj.onc.1210622 17599043

[B3] Yang M Yuan F Li P Chen Z Chen A Li S Hu C Interferon regulatory factor 4 binding protein is a novel p53 target gene and suppresses cisplatin-induced apoptosis of breast cancer cells Mol Cancer 2012 11 54 10.1186/1476-4598-11-54 22888789 PMC3447665

[B4] Klos KS Wyszomierski SL Sun M Tan M Zhou X Li P Yang W Yin G Hittelman WN Yu D ErbB2 increases vascular endothelial growth factor protein synthesis via activation of mammalian target of rapamycin/p70S6K leading to increased angiogenesis and spontaneous metastasis of human breast cancer cells Cancer Res 2006 66 4 2028 2037 10.1158/0008-5472.CAN-04-4559 16489002

[B5] Veale D Ashcroft T Marsh C Gibson GJ Harris AL Epidermal growth factor receptors in non-small cell lung cancer British J Cancer 1987 55 5 513 516 10.1038/bjc.1987.104 PMC2001730 3038157

[B6] Timchenko NA Welm AL Lu X Timchenko LT CUG repeat binding protein (CUGBP1) interacts with the 5’ region of C/EBPbeta mRNA and regulates translation of C/EBPbeta isoforms Nucleic Acids Res 1999 27 22 4517 4525 10.1093/nar/27.22.4517 10536163 PMC148737

[B7] Chang ET Donahue JM Xiao L Cui Y Rao JN Turner DJ Twaddell WS Wang JY Battafarano RJ The RNA-binding protein CUG-BP1 increases survivin expression in oesophageal cancer cells through enhanced mRNA stability Biochem J 2012 446 1 113 123 10.1042/BJ20120112 22646166 PMC4118298

[B8] Rattenbacher B Beisang D Wiesner DL Jeschke JC Von Hohenberg M St Louis-Vlasova IA Bohjanen PR Analysis of CUGBP1 targets identifies GU-repeat sequences that mediate rapid mRNA decay Mol Cell Biol 2010 30 16 3970 3980 10.1128/MCB.00624-10 20547756 PMC2916446

[B9] Zheng Y Miskimins WK CUG-binding protein represses translation of p27Kip1 mRNA through its internal ribosomal entry site RNA Biol 2011 8 3 365 371 10.4161/rna.8.3.14804 21508681 PMC3218506

[B10] Ward AJ Rimer M Killian JM Dowling JJ Cooper TA CUGBP1 overexpression in mouse skeletal muscle reproduces features of myotonic dystrophy type 1 Hum Mol Gen 2010 19 18 3614 3622 10.1093/hmg/ddq277 20603324 PMC2928132

[B11] Kang S Koh ES Vinod SK Jalaludin B Cost analysis of lung cancer management in South Western Sydney J Med Imaging Radiat Oncol 2012 56 2 235 241 10.1111/j.1754-9485.2012.02354.x 22498199

[B12] Benjamin D Moroni C mRNA stability and cancer: an emerging link? Expert Opinion Biol Ther 2007 7 10 1515 1529 10.1517/14712598.7.10.1515 17916044

[B13] Cui YH Xiao L Rao JN Zou T Liu L Chen Y Turner DJ Gorospe M Wang JY miR-503 represses CUG-binding protein 1 translation by recruiting CUGBP1 mRNA to processing bodies Mol Biol Cell 2012 23 1 151 162 10.1091/mbc.E11-05-0456 22072795 PMC3248894

[B14] Vlasova-St Louis I Bohjanen PR Coordinate regulation of mRNA decay networks by GU-rich elements and CELF1 Curr Opin Gen Dev 2011 21 4 444 451 10.1016/j.gde.2011.03.002 PMC3146975 21497082

[B15] Jones K Timchenko L Timchenko NA The role of CUGBP1 in age-dependent changes of liver functions Ageing Res Rev 2012 11 4 442 449 10.1016/j.arr.2012.02.007 22446383 PMC3610417

[B16] Devi GR siRNA-based approaches in cancer therapy Cancer Gene Ther 2006 13 9 819 829 10.1038/sj.cgt.7700931 16424918

[B17] Jiao W Zhao J Wang M Wang Y Luo Y Zhao Y Tang D Shen Y CUG-binding protein 1 (CUGBP1) expression and prognosis of non-small cell lung cancer Clin Transl Oncol 2013 15 10 789 795 10.1007/s12094-013-1005-5 23359188

[B18] Talwar S Balasubramanian S Sundaramurthy S House R Wilusz CJ Kuppuswamy D D’Silva N Gillespie MB Hill EG Palanisamy V Overexpression of RNA-binding protein CELF1 prevents apoptosis and destabilizes pro-apoptotic mRNAs in oral cancer cells RNA Biol 2013 10 2 277 286 10.4161/rna.23315 23324604 PMC3594286

